# Tanzania’s first Marburg Viral Disease outbreak response: Describing the roles of FELTP graduates and residents

**DOI:** 10.1371/journal.pgph.0003189

**Published:** 2024-05-29

**Authors:** Ally Kassim Hussein, Rogath Saika Kishimba, Azma Ayoub Simba, Loveness John Urio, Nsiande Andrew Lema, Vida Makundi Mmbaga, Beatrice Kemilembe Mutayoba, Nelson Edwin Malugu, Devotha Leonard, Joseph Hokororo, Maria Ezekiely Kelly, Albert Paschal, Danstan Ngenzi, James Andrew Hellar, George Cosmas Kauki, Grace Elizabeth Saguti, Zabulon Yoti, Kokuhabwa Irene Mukurasi, Marcelina Mponela, George S. Mgomella, Wangeci Gatei, Issesanda Kaniki, Mahesh Swaminathan, Elias Masau Kwesi, Tumaini Joseph Nagu

**Affiliations:** 1 Tanzania Field Epidemiology and Laboratory Training Program, Dar es Salaam, United Republic of Tanzania; 2 African Field Epidemiology Network, Dar es Salaam, United Republic of Tanzania; 3 Muhimbili University of Health and Allied Sciences, Dar es Salaam, United Republic of Tanzania; 4 Tanzania Field and Laboratory Epidemiologists Association, Dar es Salaam, United Republic of Tanzania; 5 Ministry of Health, Dodoma, United Republic of Tanzania; 6 Centre of Excellence in Health Monitoring & Evaluation, Mzumbe University, Morogoro, United Republic of Tanzania; 7 National Public Health Laboratory, Dar es Salaam, United Republic of Tanzania; 8 Presidents Office, Regional Authority and Local Government, Dodoma, United Republic of Tanzania; 9 World Health Organization, Dar es Salaam, United Republic of Tanzania; 10 U.S. Centres for Disease Control and Prevention, Dar es Salaam, United Republic of Tanzania; Georgetown University, UNITED STATES

## Abstract

Viral Haemorrhagic Fever Outbreak presents a significant public health threat, requiring a timely, robust, and well-coordinated response. This paper aims to describe the roles of the Tanzania Field Epidemiology and Laboratory Training Program (TFELTP) graduates and residents in responding to Tanzania’s first Marburg Viral Disease (MVD) outbreak. We performed a secondary data analysis using a range of documents, such as rosters of deployed responders and the TFELTP graduate and resident database, to count and describe them. Additionally, we conducted an exploratory textual analysis of field deployment reports and outbreak situational reports to delineate the roles played by the residents and graduates within each response pillar. A total of 70 TFELTP graduates and residents from different regions were involved in supporting the response efforts. TFELTP graduates and residents actively participated in several interventions, including contact tracing and follow up, sensitising clinicians on surveillance tools such as standard case definitions, alert management, supporting the National and Kagera Regional Public Health Emergency Operations Centres, active case search, risk communication, and community engagement, coordination of logistics, passenger screening at points of entry, and conducting Infection Prevention and Control (IPC) assessments and orientations in 144 Health Facilities. The successes achieved and lessons learned from the MVD response lay a foundation for sustained investment in skilled workforce development. FELTP Training is a key strategy for enhancing global health security and strengthening outbreak response capabilities in Tanzania and beyond.

## Introduction

Marburg Viral Disease (MVD) is a rare but highly infectious viral disease that is associated with high mortality rates [1, 2]. Africa is prone to emerging and re-emerging pathogens due to its unique ecological and socio-economic dynamics [3]. In March 2023, Tanzania reported its first MVD outbreak [4]. The affected region, Kagera, shares borders with Uganda, Rwanda and Burundi. Furthermore, its geographical location near Lake Victoria creates the possibility of human population movement and potential reservoir migrations across borders, which could raise the potential for further disease transmission [5]. Effective MVD outbreak control requires the implementation of various interventions, including case management, surveillance, contact tracing, a well-functioning laboratory service, safe and dignified burials, as well as effective risk communication and community engagement. The outbreak response is better coordinated when there is a well-established Incident Management System and preferably an Emergency Operations Centre [6]. The success of all these interventions relies on having a skilled public health workforce [7].

The Tanzania Field Epidemiology and Laboratory Training Program (TFELTP) is a competency-based training program with the objective of strengthening capacity in field epidemiology at all levels of the health system so that events of public health importance are timely detected and effectively investigated [8, 9]. The program comprises three tiers: Frontline, Intermediate, and Advanced. Since its establishment in 2008, the TFELTP has trained 924 professionals in field epidemiology across the three programs. The majority of graduates are retained in the country and encompass Tanzania’s core global health workforce routinely deployed as surge staff for rapid public health event response.

Evaluation of International Health Regulation (IHR) core capacities during the 2016 Joint External Evaluation (JEE) revealed that Tanzania had developed capacity (score 3) for human resources to implement IHR core capacity requirements, with the presence of FELTP scoring 4 (demonstrated capacity) for Human Health and 2 (basic capacity) for Animal Health. Notably, during this assessment, TFELTP had graduated 72 epidemiologists from the Advanced Course [10].

This paper aims to describe the roles of TFELTP graduates and residents in the response to the MVD outbreak spanning from mid-March to early June 2023.

## Methods

### Design

This is a descriptive case study quantifying the emergency deployment of FELTP residents and graduates as the MVD outbreak response evolved.

### Setting

Kagera is one of Tanzania’s 31 administrative regions, consisting of eight (8) districts administered by councils. It is located in the northwestern part of the country and shares borders with several neighbouring regions and countries. To the east, Kagera is bordered by Lake Victoria, Mwanza, and Mara Regions. It is bordered to the south by Geita and Kigoma Regions. Additionally, it shares borders with Rwanda to the west, Uganda to the north, and Burundi to the southwest. The total area of the region is 35,686 square kilometres, with a significant portion, around 10,173 square kilometres, being covered by water, mainly from Lake Victoria. Bukoba Urban (Municipal Council) serves as the regional capital [11]. As of the 2022 national census, the region had a population of 2,989,299 people [12]. Agriculture, including food crops and fishing, is the main economic activity in Kagera. The region has abundant wildlife, with a national park and numerous game reserves [11].

### Description of the MVD outbreak

Rumours began circulating on 16^th^ March about a potential outbreak of Viral Haemorrhagic Fever in two villages in Bukoba District, Kagera Region, involving seven cases and five deaths. These rumours were reported through various administrative channels, with a trained community health worker reporting it as an alert in the electronic event-based surveillance system. This led to an investigation by health officials from the Kagera region and the Ministry of Health, Dodoma. Following confirmatory laboratory testing, on 21^st^ March, 2023, an official declaration was released confirming that the outbreak was caused by Marburg Virus. In the end, the outbreak involved 9 cases resulting in 6 deaths (Case Fatality Rate of 66.7%). The outbreak was declared over on 2^nd^ June 2023.

### Overview of TFELTP

FELTP was modelled after the U.S. Epidemic Intelligence Service (EIS) program founded in 1951, and is currently implemented in over 80 countries worldwide [13].

TFELTP is a partnership between the Ministry of Health (MoH), Muhimbili University of Health and Allied Sciences (MUHAS), and the National Institute for Medical Research and International Partners. Partners include the U.S. Centres for Disease Control and Prevention—U.S. President’s Emergency Plan for AIDS Relief (PEPFAR), United States Agency for International Development—President’s Malaria Initiative (USAID-PMI) and African Field Epidemiology Network (AFENET) [8]. The program is anchored within the Epidemiology and Disease Control Section of the MoH. “S1 Fig” illustrates the position of the program within the MoH.

TFELTP began in 2008 with an initial cohort of Advanced Course trainees. The Advanced Course is a 2-year full-time master’s degree program which is offered by MUHAS. Trainees (residents) spend 25% of their time in class and the other 75% in the field. MUHAS awards two master’s degree options: Masters of Science in Applied Epidemiology (Epi Track) and Masters of Science in Epidemiology and Laboratory Management (Lab Track). The Advanced course aimed to produce skilled epidemiologists at the National Level. At the time of the MVD outbreak, the program was training its 14^th^ and 15^th^ Cohorts.

The Intermediate course was established in 2016 following the need to produce skilled epidemiologists at regional level. The course was a collaboration between the MoH, the University of Washington International Training and Education Center for Health (UW I-TECH); and CDC. It was the first standardised FETP Intermediate course in Africa for public health managers at sub national level [9]. The program currently runs for 6 months. It is supported by a CDC/PEPFAR Grant through Mzumbe University. The course is yet to be institutionalised in a Government Training College/University. At the time of the MVD outbreak, the program was training its 7^th^ Cohort.

The Frontline course was established in 2015 through a partnership between MOH, CDC, and the U.S. Defence Threat Reduction Agency [14]. The frontline course runs for 3 months and involves trainees from both human and animal health sectors. It targets surveillance officers at the district/ health facility levels. The program is now being offered as a short course in collaboration with MUHAS. At the time of the MVD outbreak, the program was training its 19^th^ Cohort.

TFELTP is a member of the relevant professional networks for training in field epidemiology. This includes the Training Programs in Epidemiology and Public Health Interventions Network (TEPHINET), and AFENET [8, 15]. Membership and participation in the networks have enhanced training activities for residents through training workshops and opportunities to present at their international scientific conferences. The networks provide standards for the accreditation of FETPs. The advanced course received TEPHINET accreditation in November 2018. TEPHINET accreditation assures that the quality of the training and supervision meets or even exceeds international standards [16].

A total of 924 professionals have been trained in field epidemiology through the program’s three courses, with 186 in the Advanced course, 82 in the Intermediate course, and 656 in the Frontline course.

### Data collection

We scrutinized the rosters of deployed staff and identified names of TFELTP residents and graduates. We cross referenced their sociodemographic and other characteristics in the TFELTP resident and graduate database. The database is a Microsoft excel file which contains information on graduates and trainees from all 3 tiers. We extracted their gender, professional background, highest TFELTP tier completed, whether they were residents or graduates, cohort number, employer and workstation details. We scrutinized the daily and end of deployment reports from the residents and graduates. We counted the number of residents and graduates that supported each response pillar, and described the activities that they were involved in. We also reviewed the MVD Situational Reports that were developed and shared by the Ministry of Health and abstracted quantitative information reported under each response pillar. Data sources were accessed for research purposes between the 6^th^ and 10^th^ November 2023. Data used in this study has been attached as “S1 Data”.

### Data analysis

Data were analysed in Microsoft Excel 2021 (Microsoft Office LTSC Professional Plus 2021, Seattle WA). Descriptive statistics were summarised by frequencies and percentages. The MVD epidemic curve was drawn using the date of onset of symptoms among cases. We point out significant events on the epidemic curve. We conducted an exploratory textual analysis of the activities in which the residents and graduates participated, based on the daily and end of deployment reports, as well as situational reports. Maps showing the affected district, locations from where residents and graduates were deployed, health facilities, and other important surveillance points where residents and graduates intervened were created using Q-GIS Desktop 3.28.7 (QGIS Development Team, Switzerland). We utilised the dot map plugin from the QGIS Python Plugins Repository (Dot Map Plugin). Geographic coordinates of health facilities were sourced from the MoH—Health Facility Registry Portal (Tanzania Health Facility Registry). Information on the points of entry were obtained from the Tanzania Ministry of Home Affairs–Immigration Services Department (Tanzania Ministry of Home Affairs Immigration), and geographic coordinates were sourced from Google Maps. All shapefiles used are from openly available sources (Tanzania Shape Files) and (World Bank Official Boundaries).

### Ethical consideration

The information reported in this article represents data collected during the MVD outbreak response. An outbreak investigation is regarded as an emergency activity and was endorsed by MOH. Permission to analyze and publish this information was sought and granted by the Medical Research Coordinating Committee of the National Institute for Medical Research; Reference Number BA.126/329/01a/132. All personal information collected was treated with high confidentiality, and the presented data is anonymized.

## Results

### FELTP graduates and residents who responded

A total of 70 TFELTP graduates and residents were involved during the response. Majority (81.4%) were deployed to Kagera. Laboratory Scientists and Medical Doctors contributed an equal proportion (32.9%). More than three quarters (77.1%) of the responders were Civil Servants “Table 1”.

**Table 1:** Description of TFELTP graduates and residents involved in the MVD outbreak response– 2023.

Variable	Deployed to Kagera	Supported Remotely	Total
			Responders
	(n = 57)	(n = 13)	(n = 70)
	n (%)	n (%)	n (%)
**Gender**			
Male	36 (63.2)	5 (38.5)	41 (58.6)
Female	21 (36.8)	8 (61.5)	29 (41.4)
**Training Status**			
Graduate	38 (66.7)	13 (100.0)	51 (72.9)
Resident/Trainee	19 (33.3)	0 (0.0)	19 (27.1)
**Highest Tier FELTP**			
Advanced	45 (78.9)	13 (100.0)	58 (82.9)
Intermediate	7 (12.3)	0 (0.0)	7 (10.0)
Frontline	5 (8.8)	0 (0.0)	5 (7.1)
**Background Profession**			
Laboratory Scientist	20 (35.0)	3 (23.1)	23 (32.9)
Medicine	14 (24.6)	9 (69.2)	23 (32.9)
Environmental Health	14 (24.6)	0 (0.0)	14 (20.0)
Nurse	7 (12.3)	1 (7.7)	8 (11.4)
Animal Health/ Vet	2 (3.5)	0 (0.0)	2 (2.8)
**Employment**			
Civil Servant	47 (82.4)	7 (53.8)	54 (77.1)
Implementing Partners	5 (8.7)	5 (38.5)	10 (14.3)
CDC	2 (3.5)	0 (0.0)	2 (2.9)
WHO	1 (1.8)	1 (7.7)	2 (2.9)
Africa CDC	1 (1.8)	0 (0.0)	1 (1.4)
Freelance	1 (1.8)	0 (0.0)	1 (1.4)

TFELTP graduates and residents made up 14.9% (57/382) of the health workforce responding to the MVD outbreak on the ground.

### Where were the graduates and residents deployed from?

**Fig 1:**
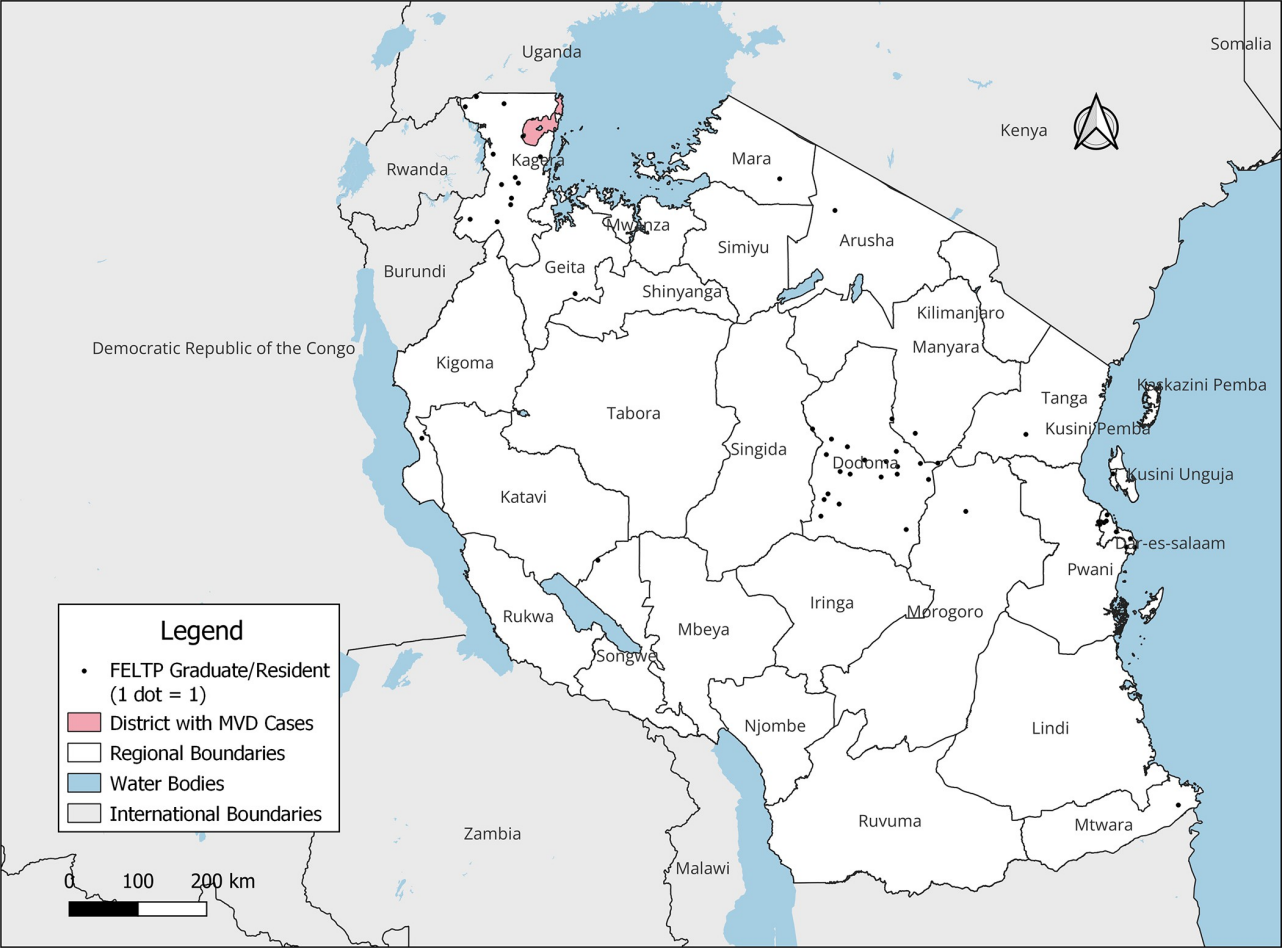
Distribution of FELTP graduates and residents that were deployed to Kagera by region of employment/ residence. This product was adapted from the NBS.

Out of the 57 Graduates and Residents that were deployed, 56 were working/residing in 13 regions of Tanzania. Majority of those deployed were from Dodoma (n = 21), Kagera (n = 15), and Dar es Salaam (n = 10). One advanced graduate was deployed by Africa CDC as a rapid responder from Uganda where he supported the recent Ebola Virus Disease Outbreak ""Fig 1"".

The shapefiles used are from openly available sources. (https://www.nbs.go.tz/index.php/en/census-surveys/gis/385-2012-phc-shapefiles-level-one-and-two) and (https://datacatalog.worldbank.org/search/dataset/0038272/World-Bank-Official-Boundaries).

Thirteen (13) of the Graduates that supported the response remotely were located in 2 Regions i.e. Dodoma (n = 7) and Dar es Salaam (n = 6).

### Deployment modalities and timeline

**Fig 2:**
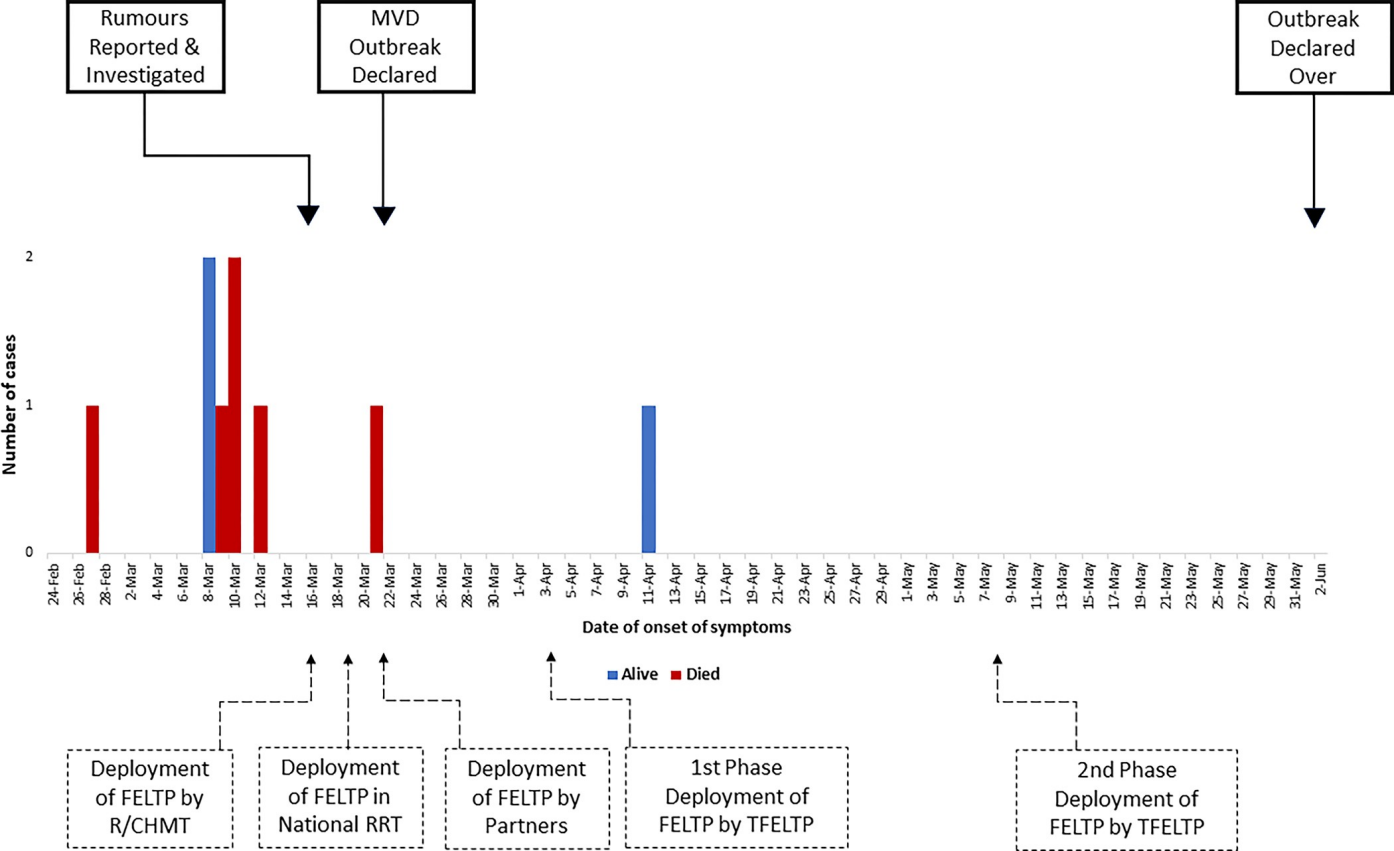
Timeline of key events and deployment patterns for TFELTP residents and graduates during the MVD outbreak.

As the graduates and residents were located in different regions and worked for different employers, several mechanisms were implemented. Graduates and residents working in Kagera were initially deployed by the Regional and Council Health Management Teams (R/CHMT) to verify the rumours. They were later joined by graduates from the Ministry of Health, Dodoma during the first days of the response as part of the National Rapid Response Team. Graduates from Implementing Partners (AMREF & MDH) and International Organizations/ Development Partners such as WHO, Africa CDC, and US CDC were deployed based on their own institutional arrangements. TFELTP and the Tanzania Field and Laboratory Epidemiologists Association (TANFLEA) mobilised residents from their field sites in Dodoma and Dar es Salaam, as well as graduates from various regions, in two phases. The first phase was from the 3^rd^ to 20^th^ of April and the second phase from the 8^th^ to 19^th^ of May ""Fig 2"".

Prior to deployment, the residents and graduates were given a two-day orientation by TFELTP staff and graduates involved in the response. The orientation covered several topics, including an overview of MVD, experiences from the Ugandan EVD response, orientation on surveillance tools, infection prevention and control for VHF, and deployment terms of reference.

### Interventions supported by TFELTP graduates and residents

#### Surveillance

**Fig 3:**
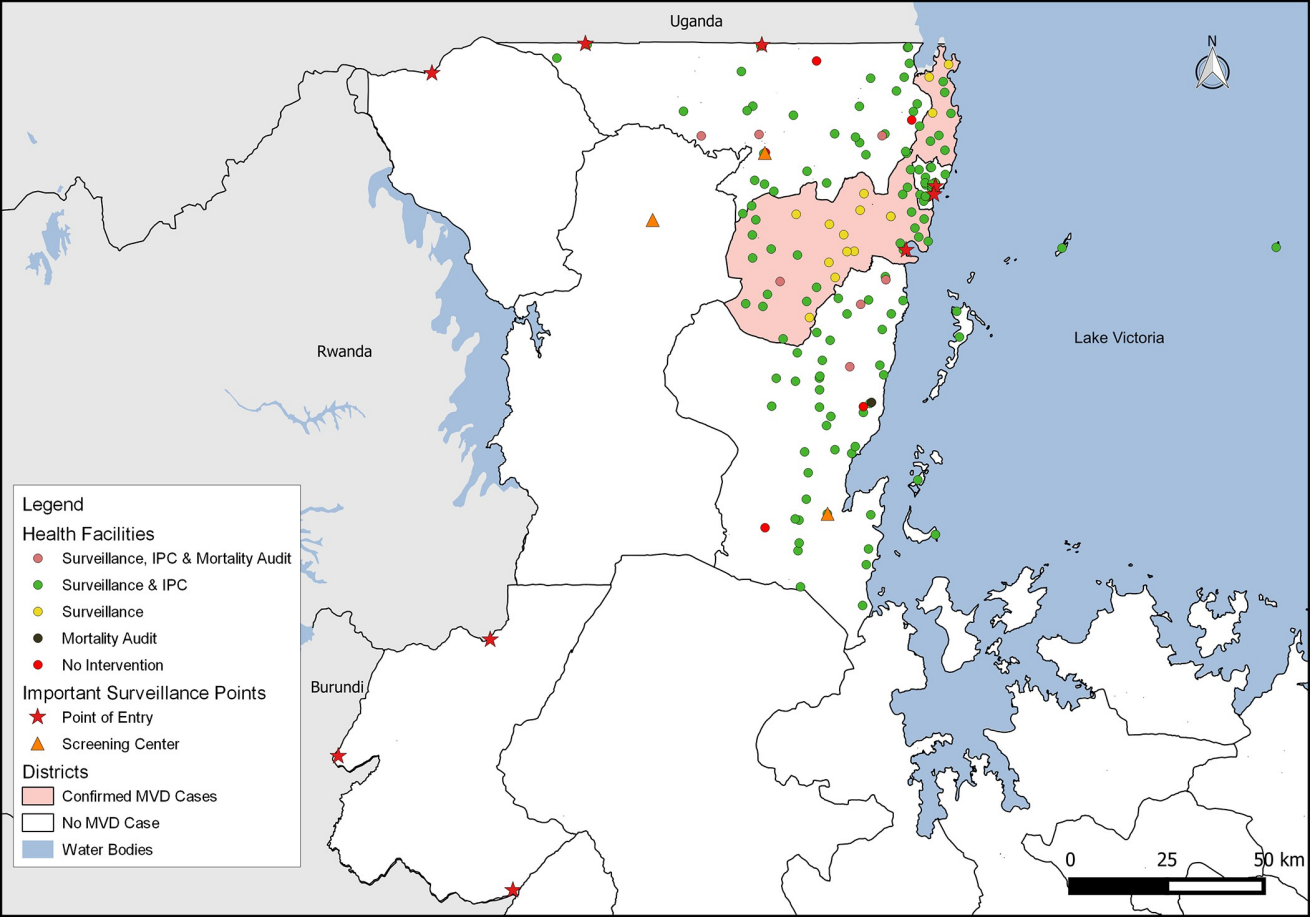
Distribution of health facilities and other important surveillance points where FELTP Graduates and Residents intervened -Kagera. This product was adapted from the NBS.

This is the main intervention/ response pillar that the graduates and residents supported ""Fig 3"".

The shapefiles used are from openly available sources: (https://www.nbs.go.tz/index.php/en/census-surveys/gis/385-2012-phc-shapefiles-level-one-and-two) and (https://datacatalog.worldbank.org/search/dataset/0038272/World-Bank-Official-Boundaries).

Of the deployed residents and graduates, 47 (82.5%) supported the surveillance pillar. They were actively involved in various aspects of surveillance, including manning the alert management desk, compiling contact lists, contact tracing and daily follow up. Additionally, they oriented clinicians on surveillance tools, conducted active case searches and performed a mortality audit.

*Alert management*. At the regional Alert Management Desk, residents and graduates were assigned the responsibility of receiving alerts (notifications/signals in the context of Event-Based Surveillance) from various sources, including the community, health facilities, and community healthcare workers. Upon receiving an alert, verification was carried out to determine if it met the standard case definition. Subsequently, a Rapid Response Team (RRT) was notified for further verification at the site. Details on the Alert Verification and Report Forms were documented. To improve the alert notification mechanism, an alert flow chart outlining the process for verifying and reporting alerts was developed. The flow charts were then distributed to health facilities. Additionally, the Desk created concise cards with MVD symptoms and a phone number for reporting. These cards were distributed to communities and health facilities during active case searches. A total of 243 alerts were reported during the response.

*Contact listing*, *tracing and daily follow-up*. Residents and graduates interviewed suspected/confirmed cases, and identified, and listed possible contacts. Contacts were placed in facilities for easy follow-up, and some were given instructions to quarantine at home. Residents and graduates supported the daily follow-up of contacts throughout the 21-day quarantine period.

Out of the 212 identified contacts, 210 successfully completed the 21-day follow-up period without experiencing any symptoms. Among the two remaining contacts, one developed symptoms and tested positive for MVD, while the other contact passed away due to unrelated causes.

*Orienting clinicians on surveillance tools*. Graduates and residents also distributed and oriented healthcare workers (n = 685) on several surveillance tools. A total of 1566 surveillance tools were distributed. These included Contact Listing Forms, Alert Management Flow Charts, Case Investigation Forms, MVD ring cards, Standard Case Definition and Protocol for Handling Suspects for Marburg.

*Active case search*. Graduates and residents designed a protocol and conducted active MVD case searches in health facilities. They conducted a retrospective review of records from patients from January to April 2023 in 44 health facilities purposefully selected in Bukoba DC and 29 health facilities in Bukoba MC. They also conducted community-based active case searches through house-to-house visits (n = 602) in 49 selected villages from Maruku and Kanyangereko wards in Bukoba DC, where the MVD cases originated. In Bukoba MC, community-based active case searches covered ten (10) wards, and 187 households were also visited. No additional MVD cases were found.

*Mortality audit*. Graduates and residents conducted a mortality data review in eight (8) health facilities in three districts of Kagera. The review focused on the number of deaths, gender distribution, monthly variations, and causes of death among different age groups from January 2022 to April 2023. A total of 945 deaths were documented with no indication of a rise in deaths attributed to MVD.

#### Coordination

Residents and graduates supported the Coordination pillar by working at the National and Kagera Regional Public Health Emergency Operations Centres and provided weekly updates to the National Task Force (NTF). They contributed to the development of the daily situational reports (Sitreps). They collated, analysed, and compiled information from all response pillars to provide situational awareness to high-level management officials at the ministry, regional, and district levels as well as other important stakeholders.

Graduates and residents were involved in creating the initial Incident Action Plan (IAP) to respond to the MVD Outbreak. The IAP aimed at identifying essential resources needed for the response, defining strategic and operational objectives, and assigning tasks to different response pillars and individuals. The IAP had an operational period of three months, starting from 16^th^ March and ending on 16^th^ June, 2023.

#### Infection prevention and control

Eleven TFELTP graduates and residents supported the assessment of infection prevention and control (IPC) practices across 144 healthcare facilities in Kagera using the infection prevention and control assessment framework (IPCAF) tool on Kobo Toolbox. During the 1^st^ phase, 29 facilities were assessed. A total of 115 facilities were assessed in the 2^nd^ phase. The facilities were located in 4 councils: Bukoba DC (n = 29), Bukoba MC (n = 29), Missenyi DC (n = 36), and Muleba DC (n = 50). IPC practices were assessed in patient service points, including laboratories, outpatient departments, labour wards, theatres, reproductive and child health departments, comprehensive care centres, and general wards.

Additionally, during the 2^nd^ phase deployment, a total of 685 staff were oriented on IPC, and a total of 2801 IPC materials were distributed in 3 Councils. The IPC materials included waste segregation guidelines, Standard Operating Procedures (SOPs) for instrument disinfection, handling spillages, safe injection practices, hand sanitization, hand washing, and housekeeping. Others include a checklist for donning and doffing personal protective equipment and instructions for preparing antiseptics and disinfectants. At least 2 pieces of each tool and SOPs were distributed at every dispensary and 3 at the health Centre level and 8 at the hospital level.

#### Water, Sanitation, and Hygiene (WASH) and Points of Entry (PoE)

Three TFELTP graduates were assigned to support the WASH and PoE pillar. They made up 8.8% (3/34) of the workforce on the ground in this pillar. The pillar was responsible for various activities, such as safe burials, decontamination, enhanced screening at points of entry, and providing WASH facilities in schools and healthcare facilities. They focused on nine (9) PoEs and three (3) community screening centres, as well as primary and secondary schools, healthcare facilities, markets, and lakeshores in Bukoba District and Bukoba Municipal councils. The team collaborated with relevant personnel to identify WASH requirements and compiled a comprehensive list of necessary items for procurement. They successfully distributed the procured materials to the designated PoEs, schools, and healthcare facilities with the support of partners like UNICEF. Additionally, the team supervised the decontamination process of hotels used for quarantine and conducted assessments of WASH needs at Bukoba Hospital, proposing measures to ensure an adequate water supply and improve waste disposal infrastructure.

A total of 500,383 travellers have been screened cumulatively since the beginning of the outbreak.

#### Risk communication and community engagement

Six TFELTP graduates and residents were involved in sensitization of the community on MVD through various channels, including health facilities, key informants, and house to house visits. TFELTP graduates and residents made up 22.2% (6/27) of the technical workforce in this pillar. They worked hand in hand with 193 Community Health Workers (CHWs). The main objective of the sensitisation was to raise awareness of symptoms of MVD, preventive measures and the importance of promptly identifying and reporting potential public health events through designated hotlines.

### Ecological study

The source of the MVD outbreak remains unidentified. However, a strong hypothesis suggests that the outbreak may have resulted from exposure to contaminated bat guano used as fertilizer. Marburg virus was previously isolated from Egyptian fruit bats (*Roussettus aegyptiacus*), which are also found in Tanzania [17, 18]. Moreover, the virus was isolated from fruit bats in two locations in Southwestern Uganda, areas in close proximity to Kagera [19, 20]. The Prime Minister’s Office through the One Health Unit is conducting an ecological study to quantify the prevalence of Marburg virus in reservoirs in Kagera. Five TFELTP graduates are involved in this study. They made up 13.5% (5/37) of the technical workforce on the ground in the study. They were involved in developing the study protocol and participated in data collection by conducting focus group discussions and in-depth interviews with key informants. Additionally, they were responsible for sample collection from humans, bats and pigs.

## Discussion

We described the diverse roles performed by TFELTP graduates and residents during the MVD Outbreak Response in Kagera. This skilled workforce took on responsibilities in various response pillars, including surveillance, coordination, infection prevention and control, water, sanitation, and hygiene, points of entry, as well as risk communication and community engagement. The TFELTP graduates and residents were well-prepared and provided valuable support to the IM, RRTs, and overall response that contributed to limiting the outbreak at source with no spill overs to neighbouring countries or regions. Furthermore, they continue to investigate key pending aspects such as the source of infection and risk factors to mitigate future outbreaks.

The program has achieved success in building a competent public health workforce that is capable of effectively responding to public health events, attributed to its training approach centred around developing key competencies. Since its establishment, TFELTP has assisted the MoH in investigating and responding to over 150 diverse disease outbreaks and public health emergencies. Notable examples include the nationwide cholera outbreak (2015 to 2018), the COVID-19 pandemic, and various other outbreaks spanning diseases such as leptospirosis, anthrax, rotavirus, acute flaccid paralysis, measles, dengue, malaria and aflatoxicosis [21–24]. TFELTP graduates have also lent their expertise to international efforts, including the 2012 Ebola Outbreak in Uganda and 2014–2016 Ebola Outbreak in West Africa [10, 25].

Similarly, competencies fostered by FE(L)TPs worldwide, empower residents and graduates to respond effectively across multiple pillars. This versatility has been notably demonstrated during the COVID-19 pandemic response where FE(L)TP residents and graduates played pivotal roles [7, 26].

The ongoing rise in threats from emerging and reemerging diseases underscores the critical need for having such a skilled public health workforce [27]. These threats pose significant challenges to health security and necessitate proactive measures to prevent, detect, and respond to outbreaks effectively [28].

TFELTP prioritised the integration of a multisectoral and One Health approach to address public health challenges, training professionals from various backgrounds [29, 30]. This approach aligns with the recommendations made during the 2016 JEE. Reflecting on the residents and graduates who responded to this outbreak, we observed a composition comprising professionals from human, animal and environmental health sectors. The program has successfully continued to enhance the IHR workforce capacity in Tanzania. Following the MVD outbreak, Tanzania conducted its 2^nd^ JEE in October 2023. Utilizing the State Party Self-Assessment Reporting Tool, the country was able to maintain its scores, which were endorsed by external evaluators [31].

Based on international recommendations, Tanzania needs a minimum of 310 field epidemiologists to meet the standard of at least 1 field epidemiologist per 200,000 population [28, 32]. Achieving this target necessitates further investments in training and capacity-building. In the response effort, Kagera region deployed all available TFELTP graduates and residents within the region, including those at the Frontline, Intermediate, and Advanced Levels. However, these resources proved insufficient. To address the response’s demands, there was a need to mobilise TFELTP graduates and residents from other regions, organizations and National Level, primarily consisting of advanced graduates and residents. To bolster response timeliness and ensure efficient responses to public health threats, it is imperative that the President’s Office, Regional Authority and Local Government (PoRALG) and MoH strategically position a larger number of graduates at subnational levels, thereby reducing the reliance on the National Level.

The outbreak was detected after 17 days from onset of symptoms of the index case, only when there were a number of related deaths, indicating that the public health surveillance capacity at the community/facility level needs to be improved. The newly launched integrated CHW training initiative could assist in bridging this gap by enhancing event-based surveillance [33].

By investing in the surveillance workforce, Tanzania can enhance its ability to identify and respond promptly to emerging and reemerging diseases. This will not only improve the country’s health security but also contribute to regional and global efforts in preventing the spread of infectious diseases and protecting public health.

TFELTP residents and graduates encountered various challenges during the response, including logistical and transportation obstacles. The program managed to address these by mobilizing vehicles from other departments of MoH and Partners, as well as securing funds for fuel to facilitate team travel. Additionally, delays in deployment especially in the 2^nd^ phase occurred due to late printing of training materials on IPC and surveillance. Development partners and implementing partners were able to resolve this issue by supporting the printing expenses. Contact listing and daily follow ups were not conducted through an electronic system, complicating matters, particularly in remote areas that were difficult to access. Collaborating with CHWs was essential in these contexts. Moreover, some contacts failed to adhere to isolation protocols, and some even absconded. Engaging community leaders and security officials enabled to trace such contacts effectively.

The limitations of this work arise from our reliance on secondary data, which hindered our ability to directly attribute accomplishments to TFELTP graduates and residents, as person-hours (time tracking/ activity logs) were not documented during the response. However, we were able to utilize multiple sources of information in the exploratory textual analysis to provide a description of the roles played by FELTP graduates and residents in the response, thereby enhancing awareness of their capabilities among others. We recommend that future efforts to describe contributions in outbreak responses include primary data collection during and immediately after deployment. This approach would ensure that every responder documents evidence of their contribution, thereby providing a more comprehensive understanding of their impact.

## Conclusion

TFELTP graduates and residents played diverse roles in responding to Tanzania’s First Marburg Viral Disease Outbreak. By nurturing a skilled workforce equipped to address evolving public health challenges, Tanzania can safeguard the health and well-being of its population and contribute to global efforts in disease prevention and control.

## Supporting information

S1 Data (XLSX)

S1 Fig Position of TFELTP within the MoH structure. (TIF)
