# Supplementary figures and images for: Tanzania’s first Marburg Viral Disease outbreak response: Describing the roles of FELTP graduates and residents
Source: PLOS Glob Public Health. 2024 May 29;4(5):e0003189. doi: 10.1371/journal.pgph.0003189 (PMC11135762; doi:10.1371/journal.pgph.0003189)

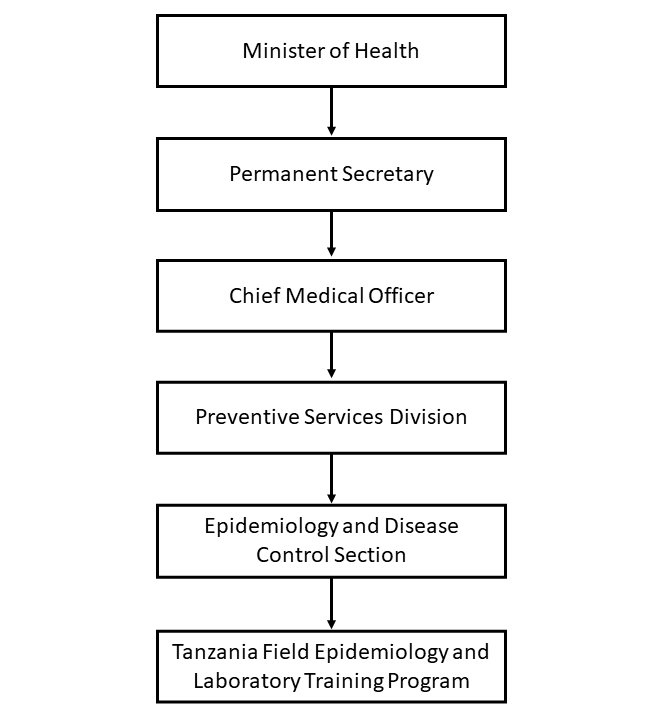

Supplement: S1 Fig — (TIF) [file pgph.0003189.s002.tif]
